# Functional Diversity and Evolution of the *Drosophila* Sperm Proteome

**DOI:** 10.1016/j.mcpro.2022.100281

**Published:** 2022-08-17

**Authors:** Martin D. Garlovsky, Jessica A. Sandler, Timothy L. Karr

**Affiliations:** 1 Department of Applied Zoology, Faculty Biology, Technische Universität Dresden, Dresden, Germany; 2 Biosciences Mass Spectrometry Core Research Facility, Knowledge Enterprise, Arizona State University, Tempe, Arizona, USA; 3 ASU-Banner Neurodegenerative Disease Research Center, The Biodesign Institute, Arizona State University, Tempe, Arizona, USA

**Keywords:** Spermatozoa, seminal fluid proteins, ribosomes, meiotic sex chromosome inactivation, fertility, evolution, discovery proteomics, human disease, OMIM, *Drosophila*, DmSP, *Drosophila melanogaster* sperm proteome, GO, gene ontology, LFQ, label-free quantitation, Sfp, seminal fluid protein, S-Laps, sperm leucyl-aminopeptidases

## Abstract

Spermatozoa are central to fertilization and the evolutionary fitness of sexually reproducing organisms. As such, a deeper understanding of sperm proteomes (and associated reproductive tissues) has proven critical to the advancement of the fields of sexual selection and reproductive biology. Due to their extraordinary complexity, proteome depth-of-coverage is dependent on advancements in technology and related bioinformatics, both of which have made significant advancements in the decade since the last *Drosophila* sperm proteome was published. Here, we provide an updated version of the *Drosophila melanogaster* sperm proteome (DmSP3) using improved separation and detection methods and an updated genome annotation. Combined with previous versions of the sperm proteome, the DmSP3 contains a total of 3176 proteins, and we provide the first label-free quantitation of the sperm proteome for 2125 proteins. The top 20 most abundant proteins included the structural elements α- and β-tubulins and sperm leucyl-aminopeptidases. Both gene content and protein abundance were significantly reduced on the X chromosome, consistent with prior genomic studies of X chromosome evolution. We identified 9 of the 16 Y-linked proteins, including known testis-specific male fertility factors. We also identified almost one-half of known *Drosophila* ribosomal proteins in the DmSP3. The role of this subset of ribosomal proteins in sperm is unknown. Surprisingly, our expanded sperm proteome also identified 122 seminal fluid proteins (Sfps), proteins originally identified in the accessory glands. We show that a significant fraction of ‘sperm-associated Sfps’ are recalcitrant to concentrated salt and detergent treatments, suggesting this subclass of Sfps are expressed in testes and may have additional functions in sperm, *per se*. Overall, our results add to a growing landscape of both sperm and seminal fluid protein biology and in particular provides quantitative evidence at the protein level for prior findings supporting the meiotic sex-chromosome inactivation model for male-specific gene and X chromosome evolution.

## Highlights

• The sperm proteome contains unexpected proteins (*e.g.*, neuronal and yolk proteins).
• Ribosomal protein “swapping” suggest stage & development specific loading into sperm.
• Label-free quantitation and fractionation revealed seminal fluid proteins in sperm.
• Low abundance of X-linked proteins supports meiotic sex-chromosome inactivation.

## Graphical Abstract

**Figure:**
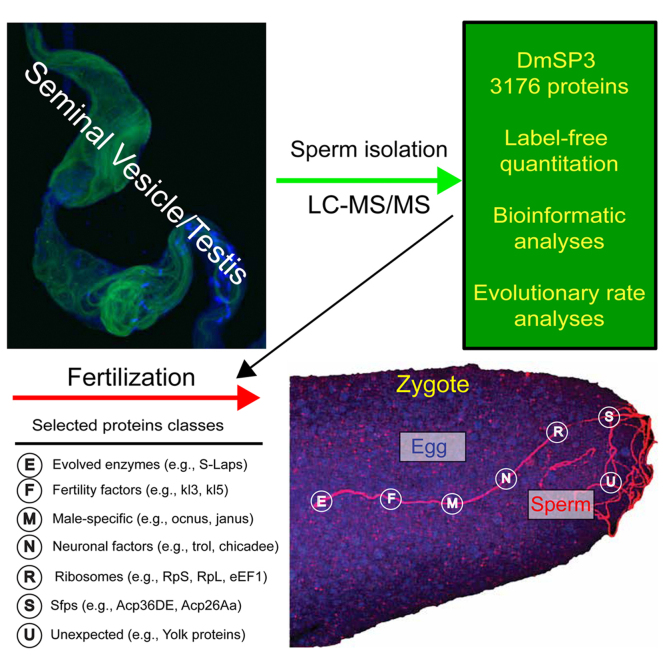

## In Brief

Fertilization is the *sine qua non* of animal and plant reproduction, a process conserved across the tree of life. During fertilization, a specific subset of male-derived proteins, many of unknown function, enter and are present in the developing egg and zygote. We therefore reinterrogated previous published sperm proteomes using improved purification and fractionation methods and identified >3000 proteins in sperm including a novel exchange of paralogous ribosomal proteins suggesting complex patterns of paralog switching and selectivity during spermatogenesis.

Spermatozoa form, function, and evolution is determined in large measure by its proteome (1). High throughput proteomics using LC-MS has been used to characterize the composition of the sperm proteome in a wide range of animals (1, 2, 3) revealing several common features of sperm as expected for an ancient cell type with a highly conserved function (4, 5). For instance, despite exhibiting exceptional morphological diversity across the tree of life (4, 6), sperm proteomes show enrichment of metabolic processes, mitochondria, axoneme, microtubules, and cytoskeletal components (2, 3, 7, 8).

*Drosophila melanogaster* provides a powerful genetic and functional genomics model system to understand reproduction and fertility (*e.g.*, (9)). Discovery (aka, shotgun) bottom-up proteomics is used to match empirical- to theoretical-peptide mass spectra and infer protein presence (10). However, although characterizing proteome composition is a critical first step, estimating protein abundance is equally critical for assigning targets of interest, potential functions, and comparative studies both within and between taxa. Indeed, the discovery and subsequent study of sperm leucyl-aminopeptidases (S-Laps) arose from protein quantitation from two-dimensional gels (11, 12). The current study was motivated by recent advances in LC-MS technology, particularly in data acquisition time and improved liquid chromatographic systems leading to enhanced proteome coverage of complex cell and tissue types (10, 13). These advances allow routine and accurate quantitation of both label- and label-free methods, an essential element for comparative studies of sperm composition and function (14, 15). Additionally, these advances permit direct injection of sample peptides without the need for prefractionation using PAGE thus avoiding sample loss, increasing proteome coverage. Here, we reinterrogated the *D. melanogaster* sperm proteome using direct solubilization of sperm followed by on-line fractionation of tryptic peptides.

Our previous efforts identified over 1000 *D. melanogaster* sperm proteins with prior versions designated DmSP1 (7) and DmSP2 (11). The DmSP3 described in this study significantly increases coverage and refinement of the *D. melanogaster* sperm proteome, from the 1108 sperm proteins identified in the DmSP2 (11) to more than 3000 proteins in the DmSP3 (Table 1). Table 1 highlights our extended knowledge base not only in terms of absolute numbers of sperm proteins but also discovery of new protein groups. We report a significant increase in both proteome size and content and provide a detailed analysis of relative abundance of sperm proteins for the first time. We confirmed high abundances of S-Laps and provide a wealth of new quantitative information including the surprising findings of substantial levels of ribosomal proteins, seminal fluid proteins (Sfps), and Y-linked proteins.

**Table 1:** History of the *Drosophila melanogaster* sperm proteome (DmSP)

Category	DmSP1	DmSP2	DmSP3
Methods/technology	LC-MS2/Maldi	SDS-PAGE	Cell digest
Machine	Thermo LCQ	LTQ Orbitrap	Orbitrap Fusion Lumos
Proteins identified	341	1108 (+767)	3176 (+2068)
X-linked^a^	Under	Ns	Under
Y-linked	0	4	9 (+5)
Sfps	CG2918	11 (+10)	122 (+111)
Ribosomal proteins	0	9	83 (+74)

DmSP1: (7); DmSP2: (11); DmSP3: this study. Note that the DmSP2 combined the 341 proteins identified in the DmSP1 with the 956 proteins identified in the DmSP2. Likewise, the DmSP3 reported here represents the combined total of all proteins identified in the DmSP2 (n = 1108) with the 2562 proteins identified across all experiments in the current study. Numbers in parentheses denote number of newly identified proteins.

a Under = significant gene underrepresentation compared to expected value (see Experimental procedures); ns = not significant.

The function of sperm beyond a delivery system for the male haploid genetic material to the next generation has gained renewed attention (16, 17, 18). For instance, sperm were often thought to be stripped of most cellular machinery, remaining transcriptionally silent prior to maturation, thus precluding the need for cellular components such as ribosomes. Additionally, previous studies found some Sfps bind to sperm in the female reproductive tract (19) and several Sfps were identified in the DmSP1 and DmSP2 but not quantified (7, 11). Therefore, we conducted two experiments to determine the binding properties of proteins associated with sperm. First, we washed sperm with a strong anionic detergent to disrupt the plasma membrane to strip away both weakly binding and sperm plasma membrane proteins. Second, we washed sperm with high molar salt to weaken ionic bonds and eliminate nonspecific protein binding to sperm (including Sfps). This approach identified over 60 ‘sperm-associated Sfps’ recalcitrant to detergent or salt treatment. Furthermore, while the current article was under review, another study also demonstrated Sfps bind sperm in the seminal vesicles (20). Therefore, we performed additional analyses to compare our results, which together provide strong evidence in support of a subclass of sperm-associated Sfps. Finally, we use the increased proteome coverage and quantitative information in the DmSP3 to provide a detailed analysis of relative abundance of sperm proteins for the first time and re-examine the evolutionary dynamics, gene age, and chromosomal distribution of sperm proteins. The analyses provide stronger support for previous claims and in particular cements the subjective prior findings supporting the meiotic sex-chromosome inactivation model for male-specific gene and X chromosome evolution (21, 22, 23, 24).

## Experimental Procedures

### Fly Stocks and Sample Preparation

We used laboratory WT strain Oregon-R *D. melanogaster* virgin males, aged 5 to 7 days. All dissections and sperm isolation were performed at room temperature in freshly prepared PBS with or without protease inhibitors (HALT, Thermo Fisher). We anaesthetized flies and removed reproductive tracts with forceps under a stereo dissecting microscope as previously described (11). Briefly, each biological replicate from ten males (20 paired seminal vesicles) were prepared separately over the course of no more than 1 hour by first removing the seminal vesicles from each male reproductive tract (containing testes, seminal vesicles, and accessory glands) into a fresh drop of PBS. Sperm were then carefully removed using fine needles to a 1.5 ml microcentrifuge tube containing 1 ml PBS (on ice). Sperm were then pelleted at 15,000 rpm for 15 min at 4 °C, PBS then carefully removed and the pellet resuspended by addition of 1.0 ml PBS followed by an additional 15 min centrifugation at 15,000 rpm. The washing procedure was repeated 2× and the final pellet immediately solubilized and reduced in 25 μl of 5% SDS/50 mM TEAB containing 50 mM DTT and incubated for 10 to 15 min at 95 °C. Samples were then spun again at 15,000 rpm for 15 min at 20 °C and visually inspected to ensure no visible pellets were present. Supernatants were then removed and stored at −20 °C or immediately processed as described below.

Solubilized sperm proteins were quantified using EZQ Protein Quantitation Kit (Thermo Fisher), and 14 to 16 μg of total protein were alkylated using 40 mM final concentration of freshly prepared iodoacetamide (Pierce) for 30 min in the dark at room temperature. Samples were processed using the S-trap Micro Columns (Protifi) following manufacturer’s S-trap Micro High Recovery Protocol. Briefly, samples (∼30 μl) were acidified to ∼1.2% phosphoric acid by addition of a stock 12% phosphoric acid solution. Proteins were digested by addition of 2 μl of a 1 mg/ml solution of porcine (MS sequencing grade modified trypsin, Promega) and layered onto the S-trap column containing 180 μl of 90% methanol/100 mM TEAB. Samples were briefly spun to remove excess buffer and washed 4× with S-trap buffer. An additional 0.5 μg of trypsin and 25 μl of 50 mM TEAB was added to the top of each column and incubated for 1 h at 47 °C. Samples were eluted off the S-trap columns using three elution buffers: 50 mM TEAB, 0.2% formic acid in water, and 50% acetonitrile/50% water +0.2% formic acid. Samples were dried down *via* speed vac and resuspended in 20 to 30 μl of 0.1% formic acid.

### Liquid-Chromatography Tandem Mass Spectrometry

All LC-MS analyses were performed at the Biosciences Mass Spectrometry Core Facility (https://cores.research.asu.edu/mass-spec/) at Arizona State University. All data-dependent mass spectra were collected in positive mode using direct-injection into an Orbitrap Fusion Lumos mass spectrometer (Thermo Scientific) coupled with an UltiMate 3000 UHPLC (Thermo Scientific). One microliter of peptides were fractionated using an Easy-Spray LC column (50 cm × 75 μm ID, PepMap C18, 2 μm particles, 100 Å pore size, Thermo Scientific). Electrospray potential was set to 1.6 kV and the ion transfer tube temperature to 300 °C. The mass spectra were collected using the “Universal” method optimized for peptide analysis provided by Thermo Scientific. Full MS scans (375–1500 m/z range) were acquired in profile mode with the Orbitrap set to a resolution of 120,000 (at 200 m/z), cycle time set to 3 s, and mass range set to “Normal”. The RF lens was set to 30% and the AGC set to “Standard”. Maximum ion accumulation time was set to “Auto”. Monoisotopic peak determination was set to “peptide” and included charge states 2 to 7. Dynamic exclusion was set to 60s with a mass tolerance of 10 ppm and the intensity threshold set to 5.0e3. MS/MS spectra were acquired in a centroid mode using quadrupole isolation window set to 1.6 (m/z). Collision-induced fragmentation energy was set to 35% with an activation time of 10 milliseconds. Peptides were eluted during a 240-min gradient at a flow rate of 0.250 μl/min, containing 2 to 80% acetonitrile/water as follows: 0 to 3 min at 2%, 3 to 75 min at 2 to 15%, 75 to 180 min at 15 to 30%, 180 to 220 min at 30 to 35%, 220 to 225 min at 35 to 80%, 225 to 230 at 80%, and 230 to 240 at 80 to 5%.

### Label-Free Quantification

We analyzed raw files searched against the Uniprot (www.uniprot.org) *D. melanogaster* database (Dmel_UP000000803.fasta) using Proteome Discover 2.4 (Thermo Scientific). Raw files were searched using SequestHT that included Trypsin (specific) as enzyme, maximum missed cleavage site 3, min/max peptide length 6/144, precursor ion (MS1) mass tolerance set to 20 ppm, fragment mass tolerance set to 0.5 Da, and a minimum of one peptide identified. Carbamidomethyl (C) was specified as fixed modification, and dynamic modifications set to acetyl and Met-loss at the N-terminus, and oxidation of Met. A concatenated target/decoy strategy and a false-discovery rate set to 1.0% was calculated using Percolator (25). The data was imported into Proteome Discoverer 2.4, and accurate mass and retention time of detected ions (features) using Minora Feature Detector algorithm. The identified Minora features were then used to determine area-under-the-curve of the selected ion chromatograms of the aligned features across all runs and relative abundances calculated.

### Gene Ontology Enrichment

We performed gene ontology (GO) enrichment analyses using the website version of DAVID (v6.8) (26). We uploaded gene lists to DAVID (https://david.ncifcrf.gov/tools.jsp) and saved outputs for all three GO categories (biological process (BP), cellular components, molecular functions) and associated statistical values. We used the default *D. melanogaster* gene list as background in DAVID to identify enriched GO terms associated with the DmSP3 (foreground n = 3176). To identify enriched GO terms associated with specific classes of sperm proteins (foreground n detailed in the results), we used the DmSP3 (n = 3176) as the appropriate background for enrichment tests. We performed network comparisons between the DmSP2 and DmSP3 using the ClueGO plugin v2.5.8 (27) for Cytoscape (v3.9.0) (28) using the default *D. melanogaster* genome background to generate enriched GO categories using a right-sided hypergeometric test and *p*-values, adjusted using Benjamini-Hochberg for multiple testing correction. Enriched GO categories with false-discovery rate values below 1% are reported. Specific parameters details are found in the figure legends.

### Evolutionary Rates

We calculated the rate of nonsynonymous (dN) to synonymous (dS) nucleotide substitutions (dN/dS) for *D. melanogaster* genes using an existing pipeline (29). We downloaded amino acid sequences and coding sequences for *D. melanogaster* (BDGP6.32) and coding sequences for *Drosophila* *sechellia* (dsec_r1.3), *Drosophila* *simulans* (ASM75419v3), and *Drosophila* *yakuba* (dyak_caf1) from Ensembl (30). For each species, we identified the longest isoform of each gene and identified orthologs using reciprocal BLASTn (31), with a minimum 30% identity and 1 × 10^−^^10^ E-value cut-off. We identified reciprocal 1:1 orthologs between all four species by the highest BLAST score and identified open reading frames using BLASTx. We then aligned orthologs using PRANK (32) and masked poorly aligned reads with SWAMP (33) using a minimum sequence length = 150, nonsynonymous substitution threshold = 7, and window size = 15. We retained 11,715 orthologs for analysis after filtering poorly aligned orthologs and those with sequence length <30 bp. We calculated one-ratio estimates (model 0) with an unrooted phylogeny: ((*D. simulans*, *D. sechellia*), *D. melanogaster*, *D. yakuba*), using the CODEML package in PAML (34), and filtered orthologs with a branch specific dS ≥ 2 or where S∗dS ≤ 1 to avoid mutational saturation. In total, we retained dN/dS estimates for 11,417 genes after filtering, including 2571 (80.95%) proteins in the DmSP3. We tested for differences in evolutionary rates between independent sets of genes using Mann-Whitney U tests.

### Experimental Design and Statistical Rationale

We designed experiments to (i) maximize proteome coverage, (ii) measure the relative abundance of individual proteins in the proteome using label-free quantitation, and (iii) examine sample purity by measuring the magnitude of adventitious protein binding and contamination in our samples. We performed three independent experiments using three treatments of purified sperm samples. In experiment 1, we collected three biological replicates of sperm in PBS only. In experiment 2, sperm were collected in either PBS and Halt protease inhibitor (“Halt” treatment), PBS only (“NoHalt” treatment), or PBS containing 0.1% Triton X100 without protease inhibitor (“PBST” treatment). In experiment 3, we collected four biological replicates of sperm prepared using either PBS (“PBS” treatment) or 2.5 M NaCl (“Salt” treatment).

We applied strict thresholds for peptide and protein identification by setting a false-discovery rate threshold at 1.0%, calculated using a reverse-concatenated target/decoy strategy in Percolator. We calculated label-free quantification (LFQ) ion intensities using the Minora feature detector in Proteome Discoverer to determine area-under-the-curve and summed technical replicates prior to analysis. To test for differences in abundance between treatments, we fit protein-wise negative binomial generalized linear models (see Statistical analysis). For experiment 2, the comparison between the Halt and NoHalt treatments was performed to determine whether active proteases were present in purified sperm samples. As only 16 proteins showed differential abundance between the Halt and NoHalt treatments (see supplemental Fig. S1), we pooled these treatments and considered them together as controls. To test the effect of detergent treatment, we subsequently performed differential abundance analysis comparing the PBST treatment to the average of both controls (Halt and NoHalt), excluding these 16 proteins (see supplementary analysis https://martingarlovsky.github.io/DmSP3/). To rank order protein abundances, we calculated a grand mean abundance for each protein across all three experiments. We excluded the PBST treatment samples from our estimates as PBST treatment significantly altered the proteome composition compared to other samples (see Results).

### Statistical Analysis

We performed all statistical analysis in R v4.03 (35). All code and analyses are available via GitHub (https://martingarlovsky.github.io/DmSP3/).

To test for nonrandom distribution of sperm proteins across the polytene chromosomes, we downloaded the chromosomal location for all genes in the genome from FlyBase.org (36) and calculated the total numbers and proportion of genes on each chromosome. We then summed the observed number of genes found in the sperm proteome on each chromosome and calculated the expected number based on the total number of sperm proteins identified by multiplying the total number of sperm proteins (n = 3176) by the expected proportion. We calculated ${\text{Χ}}^{2}$ statistics for each chromosome and the associated *p*-values with one degree of freedom and used the Benjamini-Hochberg procedure to correct for multiple testing. We excluded analysis of the Y chromosome due to the small number of protein-coding genes. To test for nonrandom distribution of sperm genes across ages classes, we downloaded gene age information from http://gentree.ioz.ac.cn/download.php (37) and grouped as ancestral (class 0; common to the *Drosophila* genus; n = 12,013), subgenus *Sophophora* (classes 1 + 2; n = 416), *melanogaster* group (class 3; n = 200), *melanogaster* subgroup (class 4; n = 334) or recent (classes 5 + 6; n = 120). We tested if sperm genes were randomly distributed across age classes compared to the rest of the genome as above, calculating the observed number of genes in each age class across the genome and among sperm proteins and calculating ${\text{Χ}}^{2}$ statistics comparing the observed *versus* expected number of genes in each age class, using the Benjamini-Hochberg procedure to correct for multiple testing.

To test for differences in protein abundance between ribosomal proteins compared to the DmSP3 average, independent groups of X-linked, Y-linked, or autosomal-proteins or between ‘high confidence’ Sfps, ‘low confidence/transferred’ Sfps *versus* remaining sperm proteins, we calculated the grand mean abundance across all three experiments excluding the PBST treatment (see Experimental design and statistical rationale). To define Sfps, we used the database compiled by Wigby *et al*. (38), who categorized ‘high confidence’ Sfps based on biochemical and bioinformatics data (n = 292) or ‘low confidence/transferred’ Sfps (n = 321) that exhibit expression or functional characteristics suggesting a potential Sfps but which require further investigation (38). We filtered proteins identified by two or more unique peptides and found in at least three biological replicates in at least one treatment group (where applicable). We performed Kruskal-Wallace rank-sum tests followed by pairwise Wilcoxon rank-sum tests corrected for multiple testing using the Benjamini-Hochberg procedure. For experiments 2 and 3, we performed differential abundance analyses using edgeR (39). For experiment 2, we filtered proteins with values in seven out of nine biological replicates. For experiment 3, we filtered to include proteins identified in at least five replicates (*i.e.*, in at least three out of four biological replicates of one treatment).

## Results

### Overview of the DmSP3

In the current study, we identified 2562 proteins across our three experiments before filtering (supplemental Fig. S2), of which 1965 (76.7%) were identified by two or more unique peptides in a single experiment (n = 1412) or in two or more replicates across any experiment (n = 1867). We obtained 106,498, 91,952, and 197,551 peptide-spectrum matches for experiments 1, 2, and 3, respectively. The numbers of peptide-spectrum matches were highly correlated between replicates within each experiment (mean Pearson’s correlation coefficient = 0.841, range 0.657–0.926, all *p* < 0.001). We measured relative protein abundances of 2125 proteins (82.9%) using LFQ. As expected from our previous study (12), α- and β-tubulins and S-Laps were among the most abundant proteins identified (Table 2). Also present were proteins of unexpected sperm prevalence including ocnus and janus B, a pair of duplicated gene products encoding a testis-specific phosphohistidine phosphatase (40), numerous Sfps, and over 80 ribosomal proteins.

**Table 2:** Most abundant proteins in the DmSP3

FBgn	Name	Chromosome arm
FBgn0003884	α-Tubulin at 84B	3R
FBgn0003889	β-Tubulin at 85D	3R
FBgn0259795	loopin-1	2R
FBgn0003885	α-Tubulin at 84D	3R
FBgn0033868	S-Lap 7	2R
FBgn0035915	S-Lap 1	3L
FBgn0052064	S-Lap 4	3L
FBgn0045770	S-Lap 3	3L
FBgn0039071	big bubble 8	3R
FBgn0034132	S-Lap 8	2R
FBgn0041102	Ocnus	3R
FBgn0031545	CG3213	2L
FBgn0037862	Mitochondrial aconitase 2	3R
FBgn0038373	CG4546	3R
FBgn0002865	Male-specific RNA 98Ca	3R
FBgn0035240	CG33791	3L
FBgn0025111	Adenine nucleotide translocase 2	X
FBgn0069354	Porin2	2L
FBgn0052351	S-Lap 2	3L
FBgn0012036	Aldehyde dehydrogenase	2L

Top 20 most abundant proteins in the DmSP3 by LFQ (rank ordered).

**Fig. 1:**
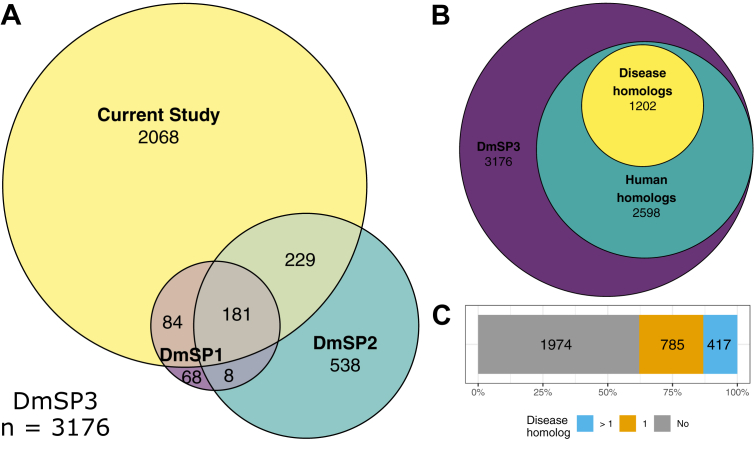
**Proteins identified in the *Drosophila melanogaster* sperm proteome.***A*, overlap between DmSP1, DmSP2, and the current study, together making up the DmSP3 (n = 3176). *B*, number of *D. melanogaster* genes found in the DmSP3 with human homologs and disease associated phenotypes from the Online Mendelian Inheritance in Man database (OMIM.org). *C*, number of *D. melanogaster* sperm protein genes with none (*gray*), one (*orange*), or more than one (*blue*) associated disease phenotype. DmSP3, *Drosophila melanogaster* sperm proteome.

Overall, we found highly consistent estimates of protein abundances between experiments. Protein abundances were strongly correlated between experiments (Pearson’s correlation = 0.86–0.89, all *p* < 0.001; supplemental Fig. S3) and median coefficients of variation for each experiment ranged from 0.018 to 0.054. We performed analyses using the entire DmSP3 (n = 3176; supplemental Table S1), combining the 2562 proteins identified in the current study with the 1108 proteins identified in the DmSP2 (7, 11) (Fig. 1*A*). While the current article was in review, another study published a list of 1409 proteins in sperm dissected from the seminal vesicles (20), the majority of which we also identified in the DmSP3 (1242/1409; 88.1%). The increased proteome coverage we achieved may be due to differences in sample preparation and experimental design (see Discussion).

### GO and Network Analyses

**Fig. 2:**
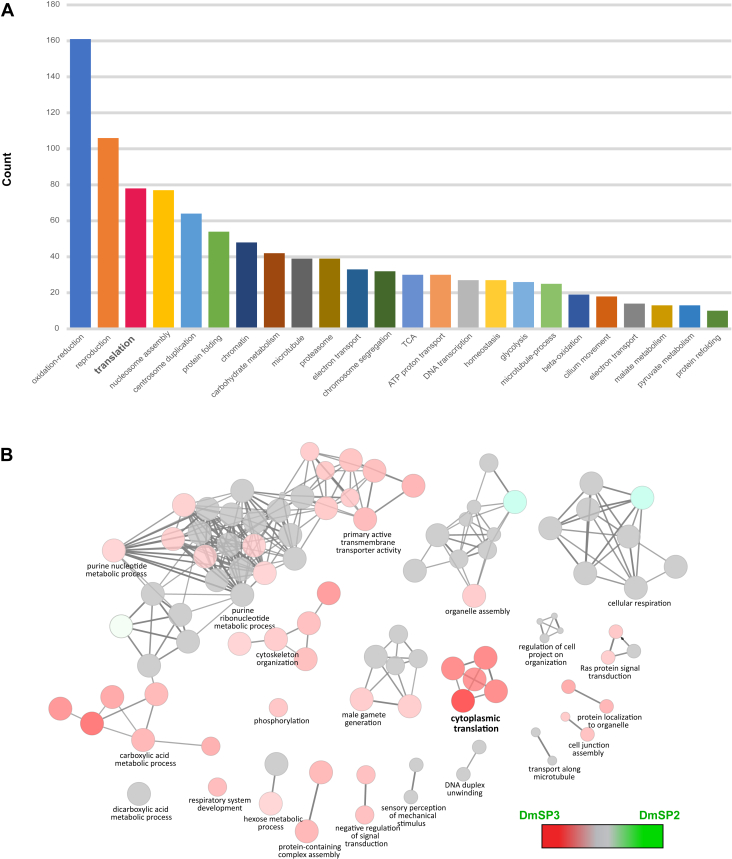
**GO functional network enrichment analysis and comparison of the DmSP2 and DmSP3.***A*, Bar graph of the 24 GO Biological Process categories identified in the DmSP3 by DAVID (26). Only functional enrichment groups with Benjamini-Hochberg corrected *p*-values < 0.01 and passing a 1% false-discovery rate threshold are shown. Note: some GO terms have been combined for clarity; see supplemental Table S2 for complete list of GO terms. *B*, GO Biological Process network comparison between the DmSP3 (3176 proteins) and DmSP2 (1108 proteins) using the ClueGO plugin for Cytoscape. Color-coded nodes within the network depict the degree of relative compositional enrichment of each dataset. The network is comprised of 22 groups (each comprised of at least 30 genes associated with a common GO functional term) containing a total of 1431 proteins. Node compositional enrichment for proteins identified in the current study (highlighted in *red*) when node composition bias exceeds 60%, while *gray* nodes indicate equal representation. *Bold* letters indicate one highly enriched category of proteins involved in cytoplasmic translation. DmSP3, *Drosophila melanogaster* sperm proteome; GO, gene ontology.

The DmSP3 is considerably larger than the DmSP2 (Fig. 1*A*), and GO analysis using DAVID identified 24 significantly enriched BP categories (Fig. 2*A* and supplemental Table S2). As expected, major categories included processes involved in energy transduction (*e.g.*, oxidation-reduction, glycolysis, TCA cycle) and reproduction. Other sperm-specific functions included terms related to microtubule and cilium movement. Surprisingly, the GO term “translation” was a prominent member in this analysis containing 78 cytosolic and mitochondrial ribosomal proteins. To further explore the GO category representation in the DmSP2 and DmSP3, we generated a heat map between the two proteomes in Cytoscape using ClueGO (Fig. 2*B*). Similar to our previous analysis of the DmSP1 and DmSP2 (11), most of the categories were equal or nearly equal in their shared properties with the one obvious exception being the aforementioned translation BP category as discussed further below.

### Human Disease Homologs

Genes in the DmSP3 are highly conserved, with 81.8% (2598/3176) having human homologs, compared to 48% of all *Drosophila* genes (41). Fully 37.8% (1202/3176) of DmSP3 genes have a homolog in humans associated with a known disease or syndrome (Online Mendelian Inheritance in Man database; OMIM.org; Fig. 1*B*). Over one third (34.7%; 417/1202) of disease associated DmSP3 genes have more than one human disease homolog (Fig. 1*C*). Among the most prevalent disease phenotypes found were susceptibility to autism, primary ciliary dyskinesia, spermatogenic failure, and myofibrillar- and congenital-myopathy (Table 3).

**Table 3:** Human disease homologs in the DmSP3

OMIM phenotype	N
Autism, Susceptibility To; AUTS20, AUTSX1, AUTSX2	27∗
Ciliary Dyskinesia, Primary; CILD40, CILD3, CILD7	25∗
Spermatogenic Failure; SPGF39, SPGF45, SPGF46	24∗
Myopathy; CFTD, MFM2, Fatal Infantile Hypertonic, Alpha-B Crystallin-Related	24∗
Hypertension, Essential	23
Type 2 Diabetes Mellitus; T2D	21
Asperger Syndrome, X-Linked, Susceptibility To; ASPGX1, ASPGX2	18∗
Cataract, Multiple Types; CTRCT16, CTRCT9	16∗
Ichthyosis, Congenital, Autosomal Recessive; ARCI4A, ARCI4B	16∗
46, XY Sex Reversal 8; SRXY8	12
Colorectal Cancer; CRC	11
Encephalopathy, Familial, With Neuroserpin Inclusion Bodies; FENIB	11
Ghosal Hematodiaphyseal Dysplasia; GHDD	10
Plasminogen Activator Inhibitor-1 Deficiency	10
Vitamin D-Dependent Rickets, Type 3; VDDR3	10
Deafness, Autosomal Recessive 91; DFNB91	9
Leukemia, Acute Myeloid; AML	9
Maturity-Onset Diabetes of The Young, Type 8, With Exocrine Dysfunction; MODY8	9
Pseudoxanthoma Elasticum; PXE	9
Cardiomyopathy, Dilated, 1II; CMD1II	8
Charcot-Marie-Tooth Disease, Axonal, Type 2F; CMT2F	8
Neuronopathy, Distal Hereditary Motor, Type IIB; HMN2B	8
Surfactant Metabolism Dysfunction, Pulmonary, 3; SMDP3	8

Most common human disease phenotypes from the Online Mendelian Inheritance in Man database (OMIM.org) associated with *D. melanogaster* genes found in the DmSP3. N = number of *D. melanogaster* genes associated with each phenotype. Similar disease phenotypes (marked with an asterisk) have been grouped. Complete list of disease associations can be found in supplemental Table S15.

### Ribosomal Proteins in the DmSP3

**Fig. 3:**
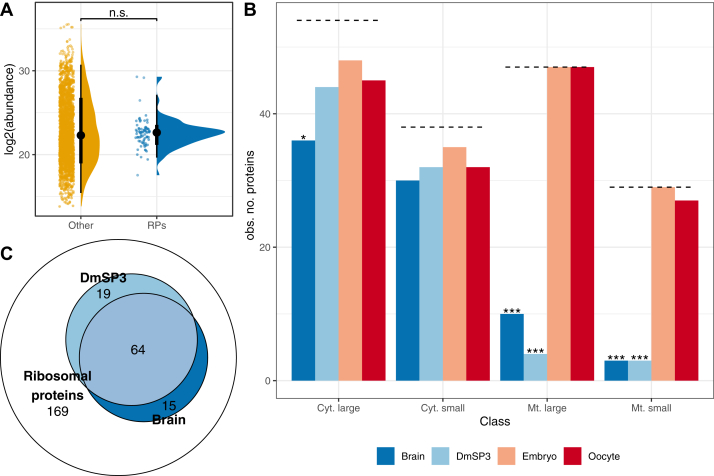
**Ribosomal proteins in the DmSP3.***A*, abundance of ribosomal proteins identified in the DmSP3 compared to the remaining sperm proteome (‘other’). Colored points represent the abundance of individual proteins. *Black points* show the mean and *thick* and *thin* bars represent the 33% and 66% confidence intervals, respectively. We compared abundances using a Kruskal-Wallace rank sum test. *B*, representation of large and small cytoplasmic and mitochondrial ribosomal proteins in the brain, DmSP3, embryo, or oocyte. The *dashed line* represents the total number of ribosomal proteins in each class and asterisks represent results from comparing the observed to expected number of proteins identified using the ${\text{Χ}}^{2}$ distribution after multiple testing correction. *C*, overlap between the total number of ribosomal proteins identified in the DmSP3 and brain tissue. n.s.; non-significant; ∗*p* < 0.05; ∗∗∗*p* < 0.001. DmSP3, *Drosophila melanogaster* sperm proteome.

Almost one-half of all *D. melanogaster* ribosomal proteins listed in FlyBase.org (83/169; 49.1%, including paralogs) were identified in the DmSP3 (supplemental Table S3). We identified the majority of cytoplasmic ribosomal proteins (76/93; 81.7%) but only 7 out of 76 (9.2%) mitochondrial ribosomal proteins. There was no significant difference in ribosomal protein abundance compared to the DmSP3 average (Kruskal-Wallis rank sum test, X^2^ = 0.063, df = 1, *p* = 0.803; Fig. 3*A*), suggesting that ribosomal proteins identified in the DmSP3 are integral to the sperm proteome and not artefactual. Furthermore, 72 of the 83 ribosomal proteins we identified were also identified by McCullough *et al*. (20).

The canonical ribosome contains 80 ribosomal proteins including 13 paralog pairs in *Drosophila* (FlyBase.org). Although the significance of paralog heterogeneity for ribosome function is currently unknown, paralog switching of ribosomal proteins has been observed in gonads and other tissues (42). We therefore compared ribosomal protein paralogs in the DmSP3 to those previously described in four tissue types including the testis (42). Significant differences were found between all four tissues as only three ribosomal protein paralogs were observed in the DmSP3 (RpL22-like, RpS14b and RpS28b) whereas both ribosomal proteins and paralog ribosomal proteins were found in all tissues with the exception of two (Rp10Aa and RpS14a; supplemental Table S4). Notably, RpL22-like is more abundant in the testis (42) whereas RpL22 was more abundant in the DmSP3. For the remaining paralogs, we identified only one member of each pair: the most abundant paralog found in the testis for seven and the less abundant paralog for two (supplemental Table S4). For RpL10Ab and RpS14b, only one paralog was identified in both the current study and Hopes *et al*. (42), and we did not identify either paralog of RpS10 (RpS10a or RpS10b). Together, these results suggest a complex landscape of paralog switching in the gonad during spermatogenesis and highlight distinct differences between sperm-ribosomal protein and testis-ribosomal protein populations.

The finding of a large number of ribosomal proteins in the DmSP3 was unexpected given sperm are thought to be transcriptionally quiescent. Therefore, we next compared the representation of ribosomal proteins found in the DmSP3 to three other recent proteomic studies in *D. melanogaster* which used Lumos Fusion Orbitrap mass-spectrometers, representing the female germline and two somatic tissues; embryo (43), unfertilized oocyte (44), and brain (45). All four tissue/cell types identified most cytoplasmic ribosomal proteins, with a slight underrepresentation of large cytoplasmic subunits in the brain (Fig. 3*B*). The DmSP3 and brain both showed significant underrepresentation of large and small mitochondrial ribosomal proteins, whereas oocyte and embryo showed almost complete representation of all ribosomal subunits (Fig. 3*B*). Significantly more ribosomal proteins identified in the brain or sperm were shared between tissues (64/98; 65.3%) than expected by chance (Fisher’s exact test, *p* < 0.001; Fig. 3*C*).

### Chromosomal Distribution of Sperm Proteins

**Fig. 4:**
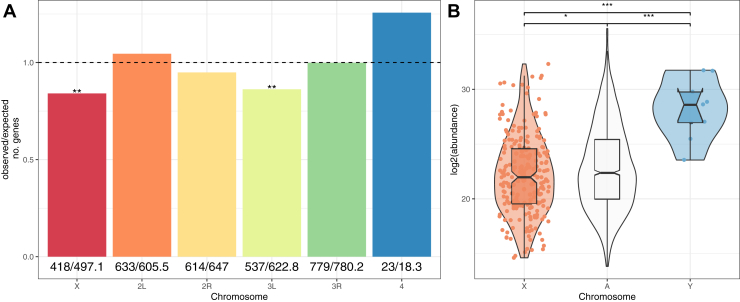
**Chromosomal distribution of DmSP3 proteins.***A*, chromosomal distribution of sperm proteins. Numbers below bars are the observed and expected number of genes on each chromosome, respectively, and the *dashed line* indicates the null expectation. Asterisks represent results from comparing the observed to expected number of genes using the ${\text{Χ}}^{2}$ distribution after multiple testing correction. *B*, abundance of sperm proteins found on autosomes (‘A’) and sex chromosomes (‘X’ or ’Y’). Points, representing individual proteins, are omitted from autosomes for clarity. Asterisks represent results from pairwise Wilcoxen rank-sum test corrected for multiple testing using the Benjamini-Hochberg procedure. n.s., non-significant; ∗*p* < 0.05; ∗∗*p* < 0.01; ∗∗∗*p* < 0.001. DmSP3, *Drosophila melanogaster* sperm proteome.

Sperm proteins were underrepresented on the X- (X^2^ = 12.6, df = 1, *p* = 0.002) and 3L- (X^2^ = 11.8, df = 1, *p* = 0.002) chromosomes (Fig. 4*A*); a pattern that was previously reported for X-linked genes in the DmSP1 (7) but not replicated in the DmSP2 (11). Protein abundance of X-linked proteins was significantly lower than those on autosomes (Mann-Whitney U test, *p* = 0.041) or the Y chromosome (Mann-Whitney U test, *p* < 0.001; Fig. 4*B*). We identified 9 of the 16 known proteins encoded on the Y chromosome (Table 4). The average abundance of Y-linked sperm proteins was higher than autosomal sperm proteins (Mann-Whitney U test, *p* < 0.001); six within the top 20% most highly abundant proteins, and all within the top 50% (Fig. 4*B* and Table 4).

**Table 4:** Y-linked sperm proteins in the DmSP3

FBgn	Name	Ranked abundance (%)	Sterile
FBgn0267433	male fertility factor kl5	98.8	Yes
FBgn0267432	male fertility factor kl3	98.8	Yes
FBgn0058064	Aldehyde reductase Y	95.5	No
FBgn0001313	male fertility factor kl2	93.6	Yes
FBgn0046323	Occludin-Related Y	92.9	No
FBgn0267449	WD40 Y	86.4	Yes
FBgn0267592	Coiled-Coils Y	86	Not studied
FBgn0046697	Ppr-Y	78.3	No
FBgn0046698	Protein phosphatase 1, Y-linked 2	65.2	No

Genes are rank ordered by mean abundance and association with male fertility from gene knockout/knockdown experiments (57, 58) are shown.

### Seminal Fluid Proteins Identified in the DmSP3

**Fig. 5:**
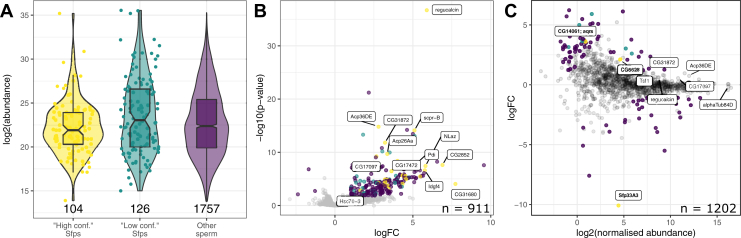
**Seminal fluid proteins in the DmSP3.***A*, log2 abundance of proteins found in the DmSP3 classified as ‘high confidence’ Sfps, ‘low confidence or transferred’ Sfps by Wigby *et al*. (38), or remaining sperm proteins. *B*, volcano plot for difference between PBST treatment *versus* the average of both controls (Halt and NoHalt) in experiment 2. Positive values indicate higher abundance in controls. *C*, MA plot for difference between NaCl treatment *versus* PBS control. Positive values indicate higher abundance in NaCl treatment. For (*B*) and (*C*), points are colored as in (*A*) denoting ‘high confidence’ (*yellow*) and ‘low confidence/transferred’ (*turquoise*) Sfps or remaining sperm proteins (*purple*) that showed significant differences in abundance based on a |logFC| > 1 and false discovery rate corrected *p*-value < 0.05. Several Sfps are labeled in (*B*) that showed differential abundance between treatments. Sfps are labeled in (*C*) that were among the top 10% most abundant proteins and the three Sfps (*bold*) that showed significant differences in abundance between treatments (*yellow*). DmSP3, *Drosophila melanogaster* sperm proteome; Sfps, seminal fluid proteins.

Sfps have been extensively studied in *Drosophila* with over 600 putative Sfps identified to date including 292 that are considered ‘high confidence’ (38). A surprisingly high number of Sfps were identified in the DmSP3 (122 ‘high confidence’ Sfps; 156 ‘low confidence/transferred’ Sfps; supplemental Table S1) (38). Another study also recently found a number of Sfps associated with sperm in the seminal vesicles, of which, we identified almost all (57/62; 91.9%) (20). We found no significant difference in abundance between Sfps and the remaining DmSP3 (Kruskal-Wallace rank-sum test, X^2^ = 4.28, df = 1, *p* = 0.118; Fig. 5*A*) and 44 ‘high confidence’ Sfps were at, or above, the median abundance of the DmSP3 (supplemental Table S5).

We therefore examined the binding characteristics of the Sfps by washing purified sperm with a strong anionic detergent (Triton X-100) known to disrupt plasma membranes. Following detergent treatment, 1600 proteins were identified, the majority (1063/1600; 66%) identified by two or more unique peptides. We identified 198 proteins that were lower abundance in PBST compared to controls (supplemental Table S6) and three proteins more abundant in PBST samples (Fig. 5*B*).

Of the 60 ‘high confidence’ Sfps identified by two or more unique peptides in experiment 2, 17 (28.4%) were filtered out prior to analysis (including 14 which were not detected in PBST samples in any replicate), and 29 (48.3%) were found at significantly lower abundance in PBST samples, together suggesting these proteins are weakly bound or found on the sperm plasma membrane. The remaining 14 (23.3%) Sfps showed no significant difference in abundance, suggesting tight association with sperm (Table 5). Additionally, 13 out of 53 (24.5%) ribosomal proteins detected in experiment 2 were significantly lower in abundance after PBST treatment. Proteins lower in abundance after PBST treatment showed GO enrichment of multicellular organism reproduction, mitochondrial transport, transmembrane transport, cytoplasmic translation, and sarcomere organization (BP). Thus, as expected, PBST treatment stripped lipids and membrane and membrane-bound proteins (including Sfps) from sperm (supplemental Table S7).

**Table 5:** Seminal fluid proteins identified in the sperm proteome after PBST treatment

FBgn	Name	Chromosome arm
FBgn0011694	Ejaculatory bulb protein II	2R
FBgn0261055	Seminal fluid protein 26Ad	2L
FBgn0004181	Ejaculatory bulb protein	2R
FBgn0003885	alpha-Tubulin at 84D	3R
FBgn0260745	midline fasciclin	3R
FBgn0036970	Serpin 77Bc	3L
FBgn0036969	Serpin 77Bb	3L
FBgn0259975	Seminal fluid protein 87B	3R
FBgn0034709	Secreted Wg-interacting molecule	2R
FBgn0264815	Phosphodiesterase 1c	2L
FBgn0020414	Imaginal disc growth factor 3	2L
FBgn0050104	Ecto-5′-nucleotidase 2	2R
FBgn0052203	Serpin 75F	3L
FBgn0003748	Trehalase	2R

In experiment 3, we washed sperm samples with high molar salt expected to weaken ionic bonds and eliminate nonspecific protein binding to sperm. We identified 1890 proteins, of which, 1273 (65%) were identified by two or more unique peptides. After filtering (see Experimental procedures), we performed differential abundance analysis for 1202 proteins and identified 92 differentially abundant proteins, including 3 Sfps (Sfp33A3, aquarius [CG14061], and CG6628) (Fig. 5*C*). The remaining 48 ‘high confidence’ Sfps we identified in this experiment did not show significant differential abundance between treatments, with six Sfps in the top 20% most abundance proteins (regucalcin, Acp36DE, CG31872, Transferrin 1, CG17097, and α-Tubulin at 84D) (Fig. 5*C*).

### Gene–Protein Abundance Concordance

To explore the relationship between protein abundance and gene expression for the 68 ‘high confidence’ Sfps tightly binding to sperm following detergent or salt treatment (‘sperm associated Sfps’; supplemental Table S8), we compared gene expression (fragments per kilobase of transcript per million mapped reads) for all proteins identified in the DmSP3 between the accessory glands, carcass, ovary, and testis using data retrieved from FlyAtlas2 (46). The average expression of both sperm-associated Sfps and the remaining Sfps identified in the DmSP3 was highest in the accessory glands, while the remaining DmSP3 proteins were most highly expressed in testis (supplemental Fig. S4*A*). However, seven sperm-associated Sfps showed higher expression in the testis than accessory glands (supplemental Table S9).

The abundance of proteins in the DmSP3 had the strongest correlation (β) and best fit (R^2^) in the testis (β = 0.460, R^2^ = 0.133, *p* < 0.001, n = 1498) (supplemental Fig. S4*B*). Protein abundance of sperm-associated Sfps was positively correlated with gene expression in the testis (β = 0.399, R^2^ = 0.152, *p* = 0.006, n = 49) but not the accessory glands (*p* = 0.246), carcass (*p* = 0.052), or ovary (*p* = 0.271). The abundance of remaining Sfps identified in the DmSP3 was positively correlated with gene expression in the accessory glands (β = 0.274, R^2^ = 0.197, *p* = 0.004, n = 41) and testis (β = 0.281, R^2^ = 0.147, *p* = 0.040, n = 29) but not the carcass (*p* = 0.109) or ovary (*p* = 0.677) (supplemental Fig. S4*C*). Therefore, our results suggest sperm-associated Sfps show tighter regulation with gene expression in the testis than accessory glands.

### Gene Age

**Fig. 6:**
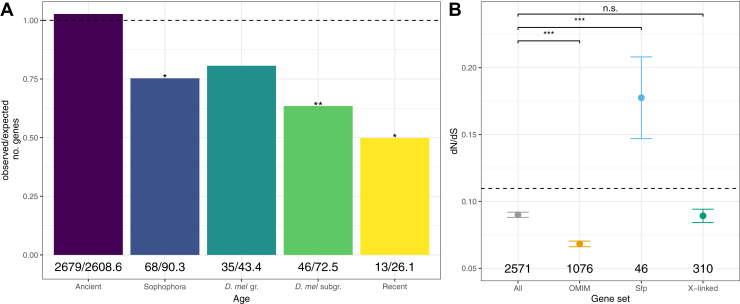
**Sperm evolutionary dynamics.***A*, gene age distribution of sperm proteins. Numbers below bars are the observed and expected number of genes in each age class, respectively, and the *dashed line* indicates the null expectation. Asterisks represent results from comparing the observed to expected number of genes using the ${\text{Χ}}^{2}$ distribution after multiple testing correction. *B*, mean (± standard error) nonsynonymous (dN) to synonymous (dS) nucleotide substitution rate (dN/dS) estimates for sperm proteins. Asterisks represent results from Mann-Whitney U tests comparing each gene set (OMIM, Sfp, X-linked) to the genome average (‘All’), excluding proteins in that set. *Dashed line* represents the genome average (mean dN/dS = 0.110, standard error = 0.001, n = 11,417). Numbers below points indicate numbers of genes in each category. Note: groups are not necessarily mutually exclusive, that is, ‘OMIM’ proteins may also be ‘X-linked’, etc. n.s., not significant; ∗*p* < 0.05; ∗∗*p* < 0.01; ∗∗∗*p* < 0.001. Sfps, seminal fluid proteins.

A variety of mechanisms drive genomic and protein diversity including gene duplication and retroposition (5, 37), resulting in unique, lineage-specific patterns of gene age (47, 48, 49). Newly evolved genes frequently acquire testis-biased gene expression (50) and it was therefore of interest to query the gene age landscape of the DmSP3. There were fewer ‘recent’ (X^2^ = 6.58, df = 1, *p* = 0.026), ‘melanogaster subgroup’ (X^2^ = 9.69, df = 1, *p* = 0.009), and ‘Sophophora-group’ (X^2^ = 5.51, df = 1, *p* = 0.032) age genes than expected by chance, indicating genes-encoding sperm proteins are underrepresented in more recent evolutionary time (Fig. 6*A*). We identified 13 genes of recent origin, of which five were located on the X chromosome and four of which are Sfps (supplemental Table S10).

### Sperm Evolutionary Rates

Genes in the DmSP3 evolve more slowly than the genome average (Mann-Whitney U test, *p* < 0.001). This pattern remains when considering X-linked sperm proteins compared to the genome average (*p* < 0.001), which evolve at a similar rate to the DmSP3 average (*p* = 0.958; Fig. 6*B*). Sfps in the DmSP3 evolve faster than the DmSP3 average (*p* < 0.001), at a similar rate to other Sfps (*p* = 0.232; supplemental Fig. S5), whereas genes with a human disease homolog (OMIM.org) evolve more slowly than the DmSP3 average (*p* < 0.001; Fig. 6*B*).

The top 10%, fastest evolving genes in the DmSP3 (dN/dS [mean ± s.e.] = 0.313 ± 0.009, n = 257, supplemental Table S11) showed GO enrichment for multicellular organism reproduction (BP) and extracellular space (cellular components) (supplemental Table S12). The bottom 10%, slowest evolving genes in the DmSP3 (dN/dS = 0.004 ± 0.0002, n = 258, supplemental Table S13), showed BP enrichment for cytoplasmic translation, centrosome duplication, regulation of cell shape, ribosomal large subunit assembly, tricarboxylic acid cycle, ATP hydrolysis coupled proton transport, cell adhesion, oocyte microtubule cytoskeleton polarization, and endocytosis (supplemental Table S14).

## Discussion

In summary, our reanalysis of the *D. melanogaster* sperm proteome (DmSP3) more than doubled the number of identified proteins, dramatically increased representation of ribosomal proteins, and highlighted several human neurological disease homologs. LFQ identified highly abundant tubulins, S-Laps, Y-linked sperm proteins, and ocnus, a testis-specific protein. LFQ also provided direct evidence for lowered abundances of X-linked sperm proteins. Sperm genes evolve relatively slowly and are underrepresented in recent age classes, consistent with evolutionary constraint acting on the sperm proteome. Finally, we identified a number of Sfps in the DmSP3, which were resistant to detergent or high molar salt treatment, suggesting some Sfps are integral to the sperm proteome.

The increased (>2-fold) depth of proteome coverage is likely due to improved protein extraction, efficiency of trypsination/peptide recovery, and direct injection methods employed in this study. Traditionally, SDS-PAGE off-line prefractionation has been the method of choice for the analysis of complex proteomes. However, these off-line methods come at a cost: sample loss due to the extra steps involved and the well-known issues of peptide recovery from polyacrylamide gels (51, 52). Although work to alleviate this limitation continues to improve this approach, our results suggest that a combination of high SDS concentrations in the initial solubilization and use of immobilized enzymatic digestion using S-Trap technologies greatly enhanced the yield of usable peptides for bottom-up proteomics. The DmSP3 also contained Yolk protein 2 (Yp2), a protein previously found in sperm (53) but undetected in the DmSP1 or DmSP2. As noted by the authors of this study (53), detection of Yp2 in sperm required large amounts of input protein for detection on immunoblots, suggesting Yp2 was present at very low levels in the testis and sperm (53). Therefore, detection of Yp2 in our study provides additional confidence in the efficacy or our approach.

The sperm proteome is expected to exhibit dynamic gene movement and expression evolution due to its sex-specific expression and essential role for male fertility (5). We found X-linked genes are underrepresented in the sperm proteome, as reported in the DmSP1 (7). Additionally, we show that X-linked sperm proteins were found in significantly lower abundances, consistent with the downstream effects of meiotic sex chromosome inactivation (22, 23, 24) and/or resolution of intralocus sexual conflict (54, 55, 56). In contrast, more than half of Y-linked proteins (9/16) including known fertility factors were present in the DmSP3 (57, 58). Our LFQ analysis revealed all nine Y-linked protein abundances were above the DmSP average, with 7/9 in the top 10%. This is the first quantitative assessment of this important class of proteins in sperm and adds direct empirical evidence in support of the long-standing hypothesized structural role in the assembly of the sperm axoneme (59).

We found sperm proteins evolve more slowly than the genome average. Slow rates of molecular evolution could be due to purifying selection or weak selection acting on sperm genes as they are shielded from selection in females (7, 60, 61). Sperm proteins were also underrepresented in recent evolutionary age classes and over 80% had human homologs, supporting the idea that sperm genes are under evolutionary constraint. A recent study found that Sfps are overrepresented in recent age classes (62), indicating different evolutionary forces acting on sperm *versus* nonsperm components of the ejaculate. Sfps in the DmSP3 evolve at a similar rate to Sfps found elsewhere in the genome and more quickly than the DmSP3 average, suggesting similar evolutionary pressures affecting rates of Sfp evolution across tissues.

The abundance of ribosomal proteins in the DmSP3 was unexpected given that sperm are stripped of most cellular machinery prior to maturation. However, sperm may undergo post ejaculatory modifications, perform secondary sexual functions, or provision the developing zygote after fertilization, requiring protein synthesis (18, 63, 64, 65). Sperm function beyond delivering a haploid compliment of nuclear material for fertilization still remains relatively underexplored (16, 17, 18). The presence of a large repertoire of core ribosomal proteins delivered to the egg during fertilization raises the intriguing possibility that paternally derived ribosomes are active during zygote formation and perhaps beyond.

Another intriguing finding that sperm had higher abundance of RpL22 *versus* the paralog RpL22-like, opposite from levels found in the testis (42), suggests a complex pattern of paralog switching and selectivity during spermatogenesis. While the functional significance of this selectivity is unknown, they are interesting to consider in the context of the known mRNA repertoire in *Drosophila* sperm delivered to the egg at fertilization (65). Fully 33% of the total sperm mRNA repertoire encoded ribosomal proteins (47/142; ref. (65)), a striking coincidence that warrants further study. We also found similarity in the underrepresentation of mitochondrial ribosomal proteins in both the DmSP3 and brain, providing yet another example of the molecular similarities between these two tissue types (66). Finally, we note that the DmSP3 contains as many as 300 entries with GO annotation terms related to neuronal structure and function, lending additional support to the similarities drawn between the brain and testis.

### Possible Testis Origin of Seminal Fluid Proteins

Although some Sfps were previously identified, but not quantified in the DmSP2 (11), the unexpectedly high numbers (and in some cases, relative abundances) of Sfps found in the DmSP3 adds to an expanding landscape of Sfp biology. As Sfps are thought to be primarily secreted from the paired accessory glands and the ejaculatory bulb in *Drosophila* (67), our results raise the possibility that some Sfps are integral to the sperm proteome and are secreted from the testes or seminal vesicles, or bind to sperm prior to mixing in the ejaculatory duct. We identified 122 ‘high confidence’ Sfps (38) in the DmSP3 which is unlikely artefactual given that many Sfps were found in multiple biological replicates and in independent experiments. An independent study conducted in another laboratory using a different *Drosophila* strain identified an almost identical list of ‘sperm-associated Sfps’ (20), thus providing strong evidence in support of a possible testis origin of some Sfps. Denaturing the sperm plasma membrane using detergent stripped most (75%) Sfps from the sperm proteome, suggesting these Sfps are integral to the sperm plasma membrane or bound to sperm advantageously in the seminal vesicles prior to mixing in the ejaculatory duct. High molar salt had little effect on the composition of the sperm proteome, indicating some Sfps are bound strongly to sperm.

We identified 68 ‘sperm-associated Sfps’ that were not depleted by detergent or salt treatment. Thus, we suggest several of the ‘high-confidence’ Sfps in the DmSP3 and also highly expressed in the testes (supplemental Table S9) should be classified as sperm proteins. In addition, α-Tubulin at 84D (FBgn0003885) is a major constituent of microtubules and involved in sperm axoneme assembly and therefore likely a sperm protein (See also supplemental Tables S1 and S2 in (20)). Notably, Acp36DE was consistently among the most abundant proteins in our experiments. Previous studies have shown Acp36DE tightly binds to sperm in the female reproductive tract after mating and is essential for efficient sperm storage in the female sperm storage organs (19, 68). The possibility that Sfps bind to sperm in the seminal vesicles prior to mixing in the ejaculatory duct should be investigated further (20). Moreover, the potential for the testes, seminal vesicles, or perhaps even sperm cells, to secrete proteins, including Sfps, requires further investigation.

Finally, the DmSP3 contains over 1200 human disease homologs. The prominence of several neurological diseases (*e.g.*, Primary Ciliary Dyskinesia, susceptibility to autism, encephalopathy, and neuropathy) may be related to the shared functional designs of sperm and neurons, cells of extraordinary axial ratios transmitting biological information over large distances. It will be of great interest to tease out the significance of this subset of neural-related DmSP3 proteins in the context of sperm function and its related reproductive activities and possible relevance for study of human diseases.

## Conclusion

Our reanalysis of the *D. melanogaster* sperm proteome using improved separation and detection methods and an updated genome annotation highlights several key features of sperm function and evolution, including the prominence of proteins integral to sperm development (tubulins and S-Laps), the dynamic nature of sex-linked sperm genes, and constraints on sperm proteome evolution. We also show the prevalence of many ribosomal proteins, despite the expectation that sperm are transcriptionally silent. The parallels in ribosomal protein composition and occurrence of several human neurological disease homologs also lend further support to the functional similarities between sperm and neurons. Finally, we demonstrate that a significant number of Sfps are found in the sperm proteome raising the possibility that Sfps mix with sperm in the seminal vesicles or Sfps may be secreted from the testes, seminal vesicles, or even sperm cells.

## Data Availability

Proteomic data have been deposited to the ProteomeXchange Consortium *via* the PRIDE partner repository (69) with the identifier PXD032033. All code and analyses are available from GitHub: https://martingarlovsky.github.io/DmSP3/

## Supplemental data

This article contains supplemental data (46).

## Conflict of interest

The authors declare that they have no conflicts of interest with the contents of this article.

## Supplemental Data

Supplemental Fig S1 Volcano plots from pairwise analyses between treatments in experiment two, denoting ‘high confidence’ (yellow) and ‘low confidence/transferred’ (turquoise) Sfps or remaining sperm proteins (purple) that showed significant differences in abundance based on a |logFC| > 1 and false discovery rate corrected p-value < 0.05. Several Sfps are labelled that showed differential abundance between treatments.

Supplemental Fig S2 Overlap between proteins identified in the current study. The central Euler diagram shows the overlap between proteins identified in each experiment. The surrounding diagrams show the overlap in number of proteins identified in each replicate of each treatment, coloured according to experiment as in the central diagram.

Supplemental Fig S3 Correlations in average protein abundance between each experiment in the current study. Mean protein abundance was calculated across all replicates for each experiment, except experiment two which excluded the PBST treatment. Shown are Pearson’s correlations and line of best fit.

Supplemental Fig S4 Gene–protein abundance concordance in the DmSP3. a) Heatmap of mRNA expression of DmSP3 genes (n = 2673) in the accessory glands, carcass, ovary, and testis. Data retrieved from from FlyAtlas2 (46) are log2(FPKM) scaled per gene. The 7 ‘high confidence’ Sfps with higher expression in the testis than accessory glands are highlighted on the right. Labels on the right also show ‘sperm associated Sfps’ (red) and other ‘high confidence’ Sfps (blue) identified in the DmSP3. b) Linear regressions of gene expression on protein abundance in the testis (n = 1498), carcass (n = 1165), accessory glands (n = 1001), and ovary (n = 825). c) Linear regressions of gene expression on protein abundance for ‘sperm associated Sfps’ (SAS) and remaining Sfps identified in the DmSP in each tissue. b) and c) are linear regressions using z-score log2-transformed values after filtering genes with log2-FPKM < 2. Blue lines are model fits from a linear regression, dashed lines indicate a perfect correlation between gene expression and protein abundance.

Supplemental Fig S5 Nonsynonymous (dN) to synonymous (dS) nucleotide substitution rate (dN/dS) estimates for proteins in the DmSP3 or elsewhere. Asterisks represent results from Mann-Whitney U tests; n.s., non-significant; ∗∗∗, p < 0.001.
