# Supplementary figures and images for: Functional Diversity and Evolution of the Drosophila Sperm Proteome
Source: Mol Cell Proteomics. 2022 Aug 17;21(10):100281. doi: 10.1016/j.mcpro.2022.100281 (PMC9494239; doi:10.1016/j.mcpro.2022.100281)

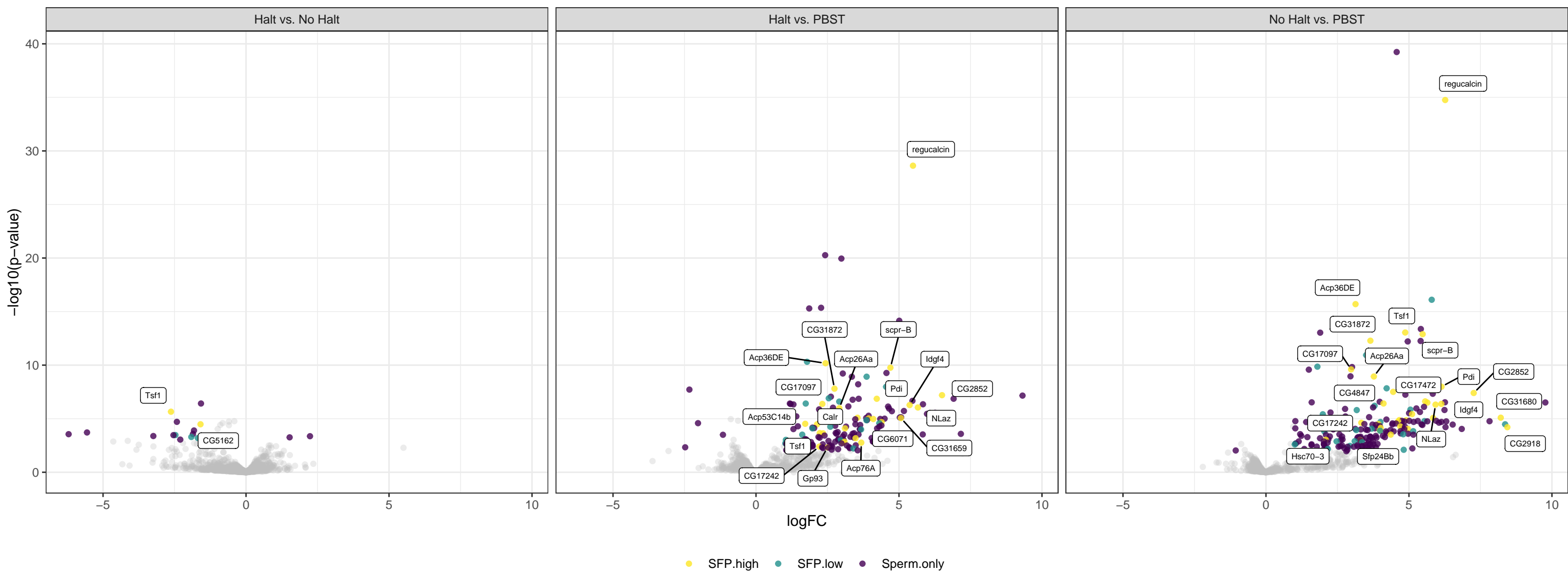

Supplement: Supplemental Fig S1 — Volcano plots from pairwise analyses between treatments in experiment two, denoting ‘high confidence’ (yellow) and ‘low confidence/transferred’ (turquoise) Sfps or remaining sperm proteins (purple) that showed significant differences in abundance based on a |logFC| > 1 and false discovery rate corrected p-value < 0.05. Several Sfps are labelled that showed differential abundance between treatments. [file mmc1.pdf]

# Experiment 3

NaCl treatment

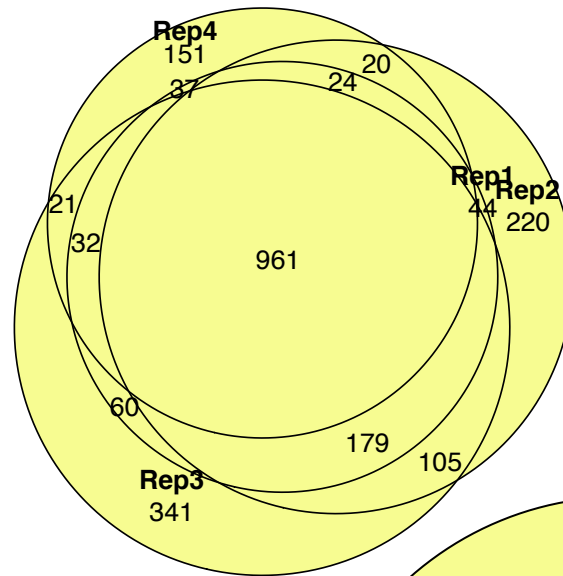

PBS control

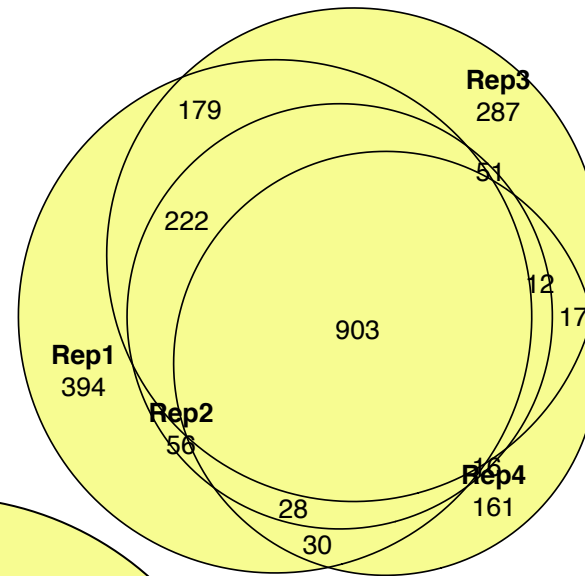

# Experiment 2

PBST

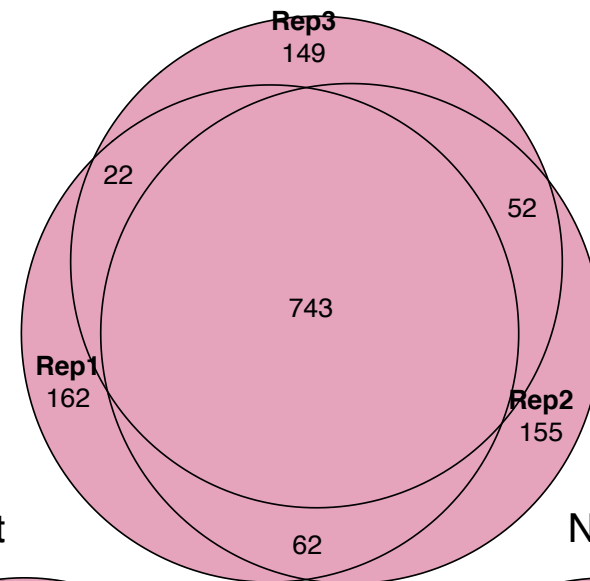

# Experiment 1

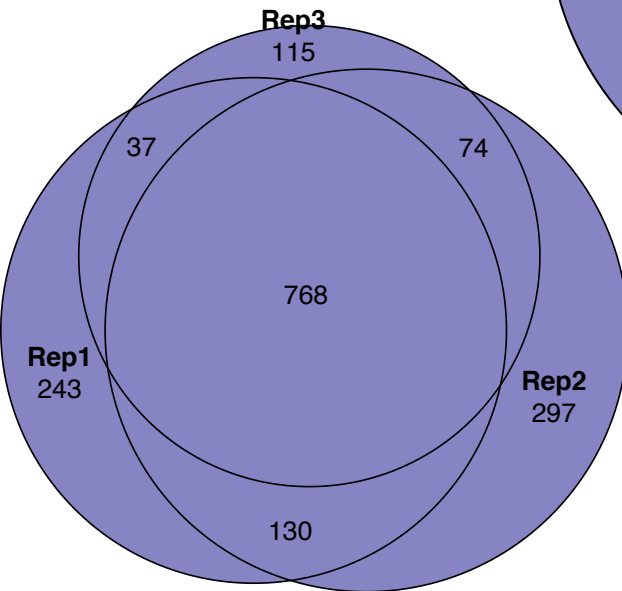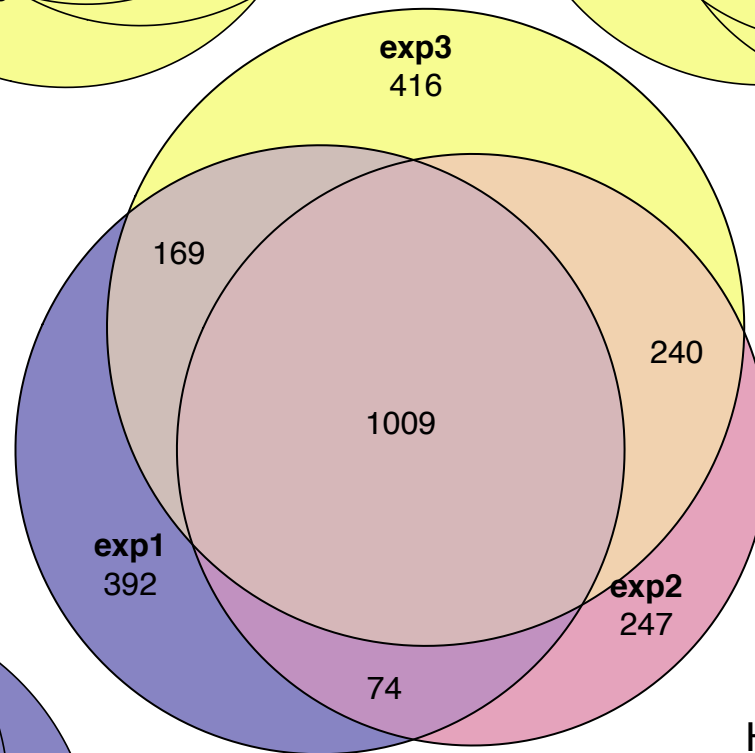

Halt

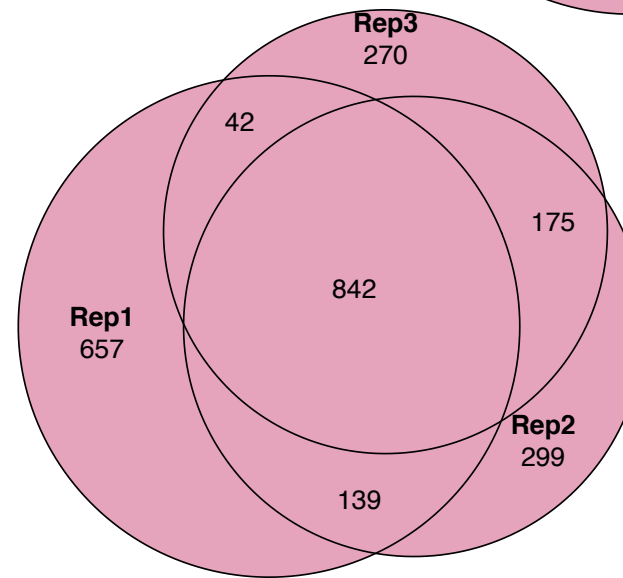

NoHalt

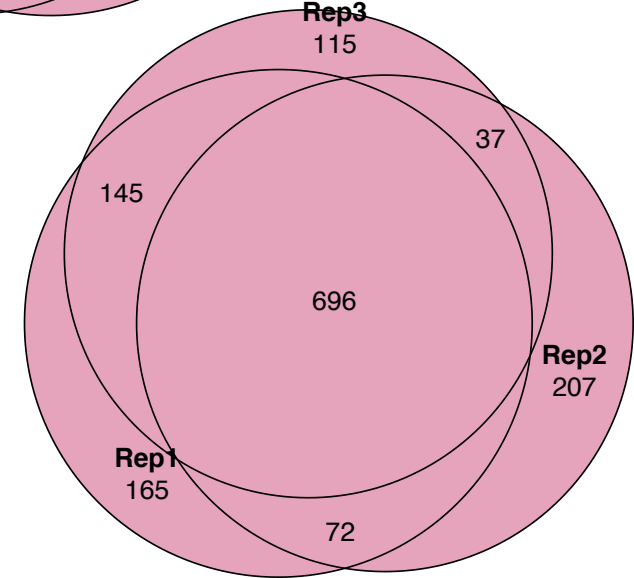

Supplement: Supplemental Fig S2 — Overlap between proteins identified in the current study. The central Euler diagram shows the overlap between proteins identified in each experiment. The surrounding diagrams show the overlap in number of proteins identified in each replicate of each treatment, coloured according to experiment as in the central diagram. [file mmc2.pdf]

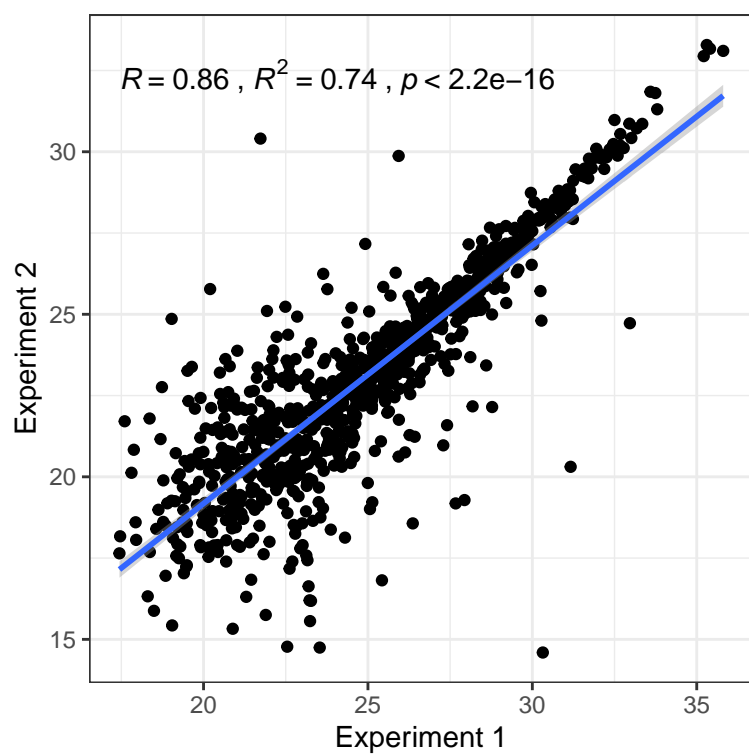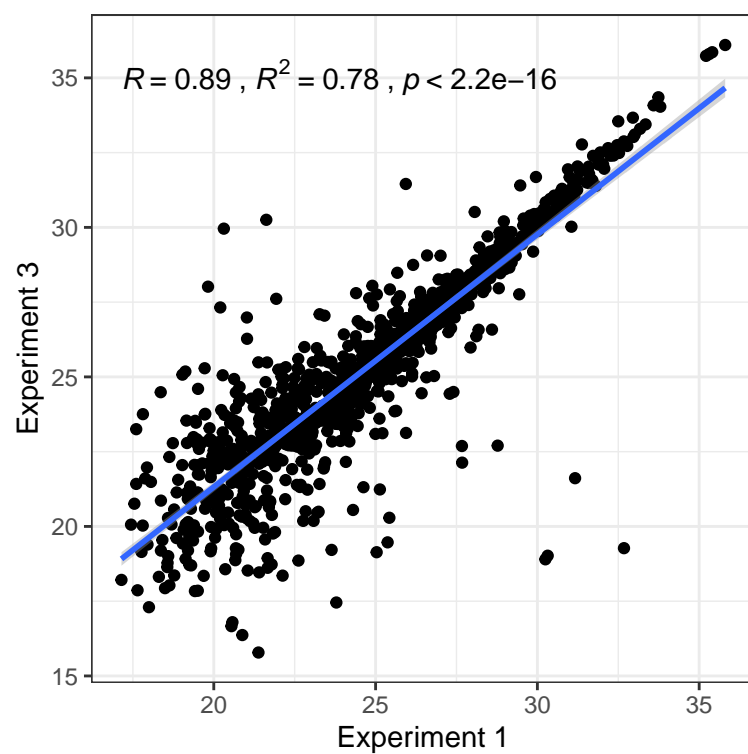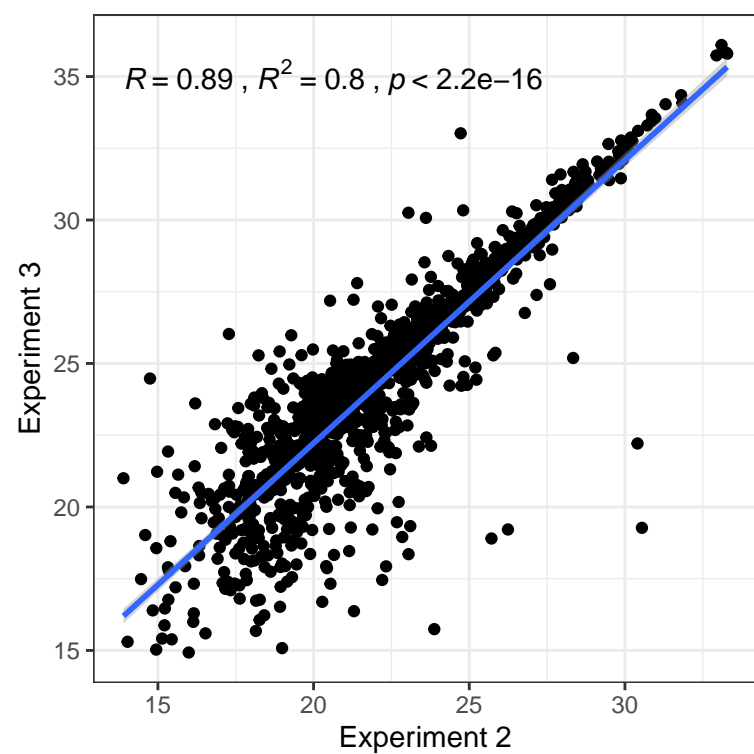

Supplement: Supplemental Fig S3 — Correlations in average protein abundance between each experiment in the current study. Mean protein abundance was calculated across all replicates for each experiment, except experiment two which excluded the PBST treatment. Shown are Pearson’s correlations and line of best fit. [file mmc3.pdf]

a)

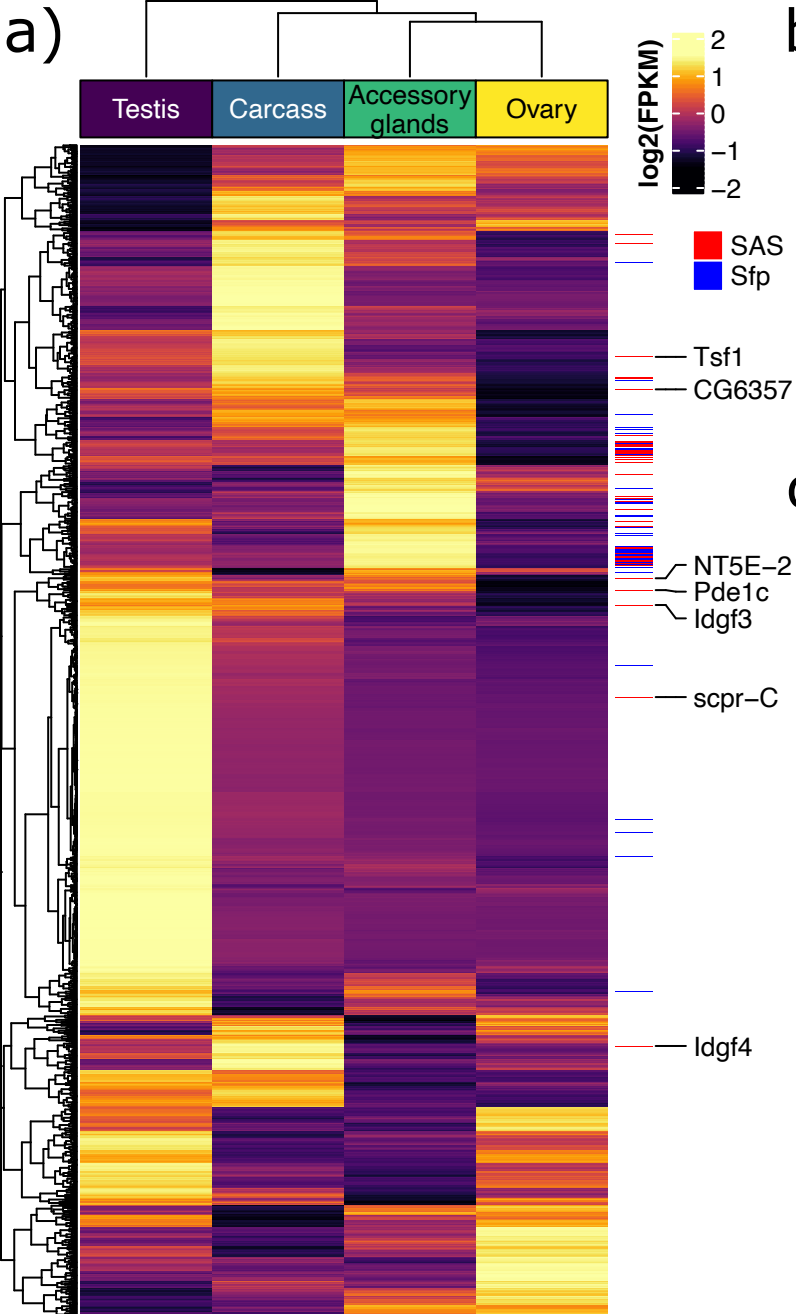

b)

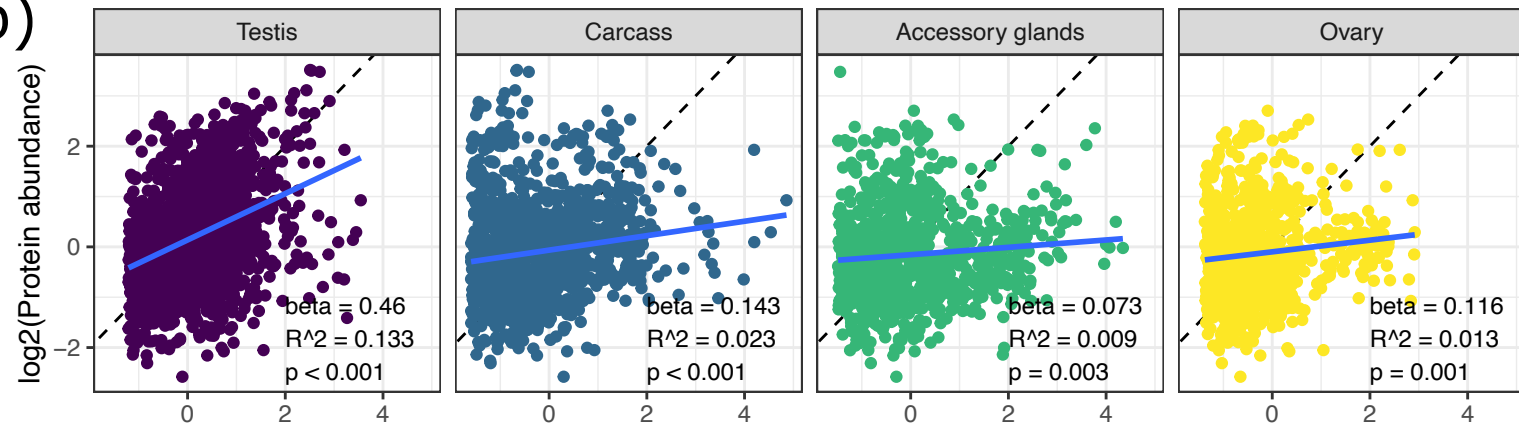

c)

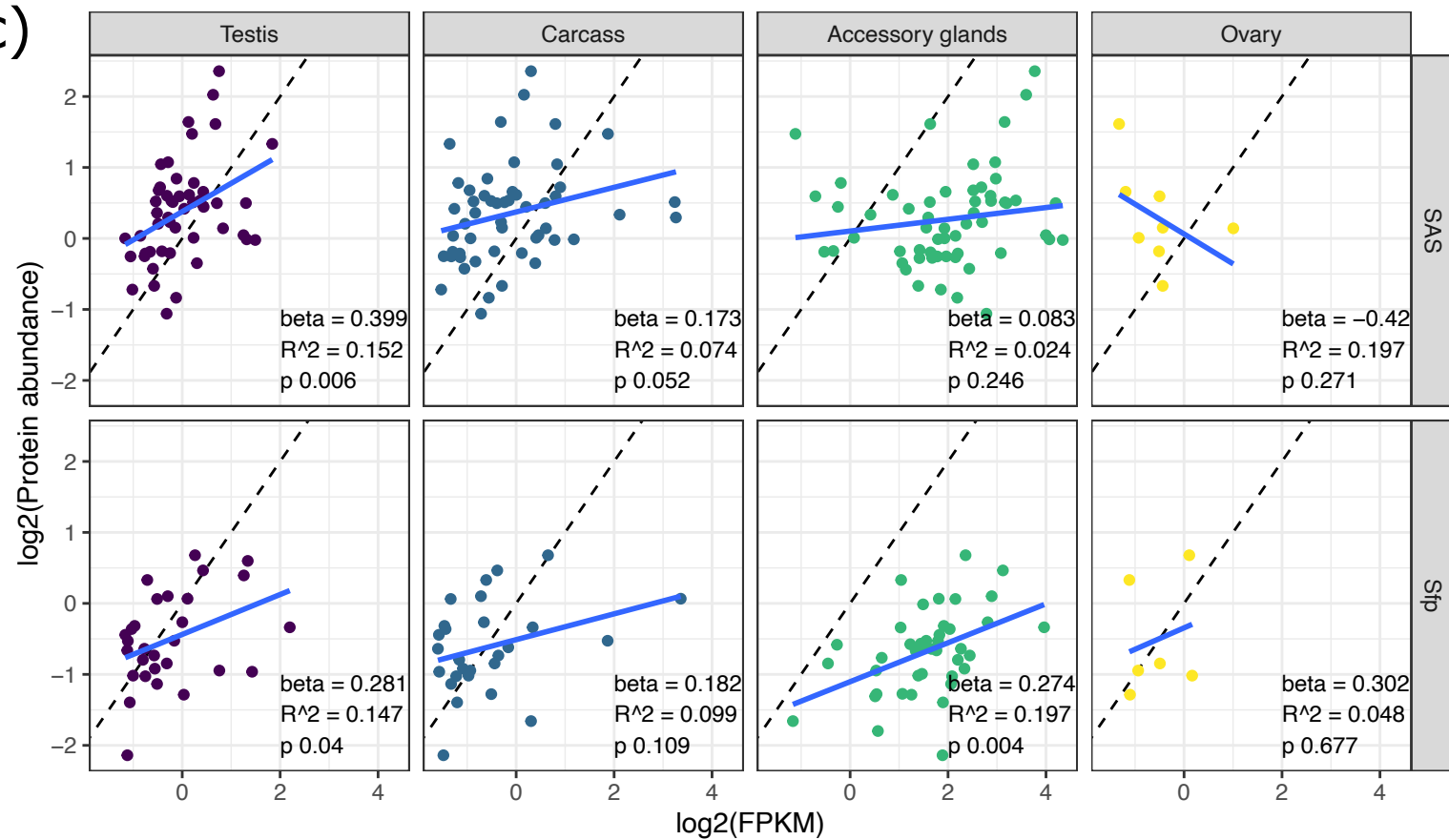

Supplement: Supplemental Fig S4 — Gene–protein abundance concordance in the DmSP3. a) Heatmap of mRNA expression of DmSP3 genes (n = 2673) in the accessory glands, carcass, ovary, and testis. Data retrieved from from FlyAtlas2 (46) are log2(FPKM) scaled per gene. The 7 ‘high confidence’ Sfps with higher expression in the testis than accessory glands are highlighted on the right. Labels on the right also show ‘sperm associated Sfps’ (red) and other ‘high confidence’ Sfps (blue) identified in the DmSP3. b) Linear regressions of gene expression on protein abundance in the testis (n = 1498), carcass (n = 1165), accessory glands (n = 1001), and ovary (n = 825). c) Linear regressions of gene expression on protein abundance for ‘sperm associated Sfps’ (SAS) and remaining Sfps identified in the DmSP in each tissue. b) and c) are linear regressions using z-score log2-transformed values after filtering genes with log2-FPKM < 2. Blue lines are model fits from a linear regression, dashed lines indicate a perfect correlation between gene expression and protein abundance. [file mmc4.pdf]

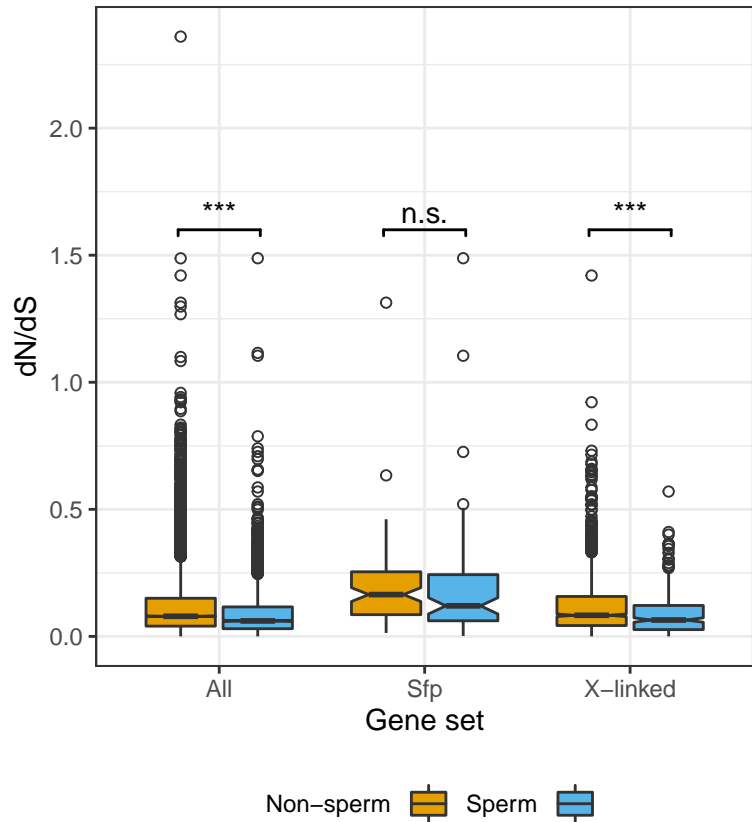

Supplement: Supplemental Fig S5 — Nonsynonymous (dN) to synonymous (dS) nucleotide substitution rate (dN/dS) estimates for proteins in the DmSP3 or elsewhere. Asterisks represent results from Mann-Whitney U tests; n.s., non-significant; ∗∗∗, p < 0.001. [file mmc5.pdf]
